# Pennyroyal

**Published:** 1891-01-24

**Authors:** 


### TOXICOLOGY.

### PENNYROYAL.

This drug, as is well known, is largely used in England and some other countries as a popular emmenogogue and, though not always ostensibly, an abortifacient. Medical opinion, however, is not agreed as to its claims to be regarded as a uterine specific, some authorities, as Taylor, considering it almost inert; and others as more dangerous than efficient. Dr. Edmond Falk, of the Pharmacological Institute, at Berlin, has, in conjunction with Dr. Langgaard, carried out a number of experimental observations on rabbits which show conclusively that it is by no means innocuous. Subcutaneous injections of one to two grams of the oil caused marked intoxication, reeling gait, and loss of co-ordination lasting some time. Three grams caused the same symptoms in a more severe form, and death in a day or two. Injections of one gram repeated daily were followed by death about the third day, with albumen, leucine, and tyrosin in the urine, granular casts, and total suppression of urine preceding the fatal termination by a few hours. Still smaller doses of ·01 gram daily did not prove fatal until the second week, but soon caused, besides the nervous phenomena in slighter degree, albuminuria with numerous casts, bloody, fatty, granular, and hyaline. Post-mortem examinations showed enlargement, fatty degeneration, and yellow staining, closely resembling that seen in phosphorus poisoning, of the heart, liver, and kidneys, in this order of intensity. He concludes from these experiments that pennyroyal has no specific action, but that the effects which have been interpreted in that light are merely owing to its general toxic influence on the internal organs.

